# A simplified and cost-effective enrichment protocol for the isolation of *Campylobacter *spp. from retail broiler meat without microaerobic incubation

**DOI:** 10.1186/1471-2180-11-175

**Published:** 2011-08-03

**Authors:** Ping Zhou, Syeda K Hussain, Mark R Liles, Covadonga R Arias, Steffen Backert, Jessica Kieninger, Omar A Oyarzabal

**Affiliations:** 1 Department of Biological Sciences, 1627 Hall Street, Alabama State University, Montgomery, AL, USA; 2 Department of Microbiology and Immunology, University of Arkansas for Medical Sciences, Little Rock, AK, USA; 3 Department of Biological Sciences, 101 Rouse Life Science Bldg Auburn University, AL, USA; 4 Department of Fisheries and Allied Aquacultures, 101 Swindle Hall, Auburn University, AL, USA; 5 University College Dublin, UCD School of Biomolecular and Biomedical Sciences, Science Center West, Belfield Campus, Dublin 4, Ireland

## Abstract

**Background:**

To simplify the methodology for the isolation of *Campylobacter *spp. from retail broiler meat, we evaluated 108 samples (breasts and thighs) using an unpaired sample design. The enrichment broths were incubated under aerobic conditions (subsamples A) and for comparison under microaerobic conditions (subsamples M) as recommended by current reference protocols. Sensors were used to measure the dissolved oxygen (DO) in the broth and the percentage of oxygen (O_2_) in the head space of the bags used for enrichment. *Campylobacter *isolates were identified with multiplex PCR assays and typed using pulsed-field gel electrophoresis (PFGE). Ribosomal intergenic spacer analyses (RISA) and denaturing gradient gel electrophoresis (DGGE) were used to study the bacterial communities of subsamples M and A after 48 h enrichment.

**Results:**

The number of *Campylobacter *positive subsamples were similar for A and M when all samples were combined (*P *= 0.81) and when samples were analyzed by product (breast: *P *= 0.75; thigh: *P *= 1.00). Oxygen sensors showed that DO values in the broth were around 6 ppm and O_2 _values in the head space were 14-16% throughout incubation. PFGE demonstrated high genomic similarity of isolates in the majority of the samples in which isolates were obtained from subsamples A and M. RISA and DGGE results showed a large variability in the bacterial populations that could be attributed to sample-to-sample variations and not enrichment conditions (aerobic or microaerobic). These data also suggested that current sampling protocols are not optimized to determine the true number of *Campylobacter *positive samples in retail boiler meat.

**Conclusions:**

Decreased DO in enrichment broths is naturally achieved. This simplified, cost-effective enrichment protocol with aerobic incubation could be incorporated into reference methods for the isolation of *Campylobacter *spp. from retail broiler meat.

## Background

Campylobacteriosis in the most common foodborne disease in European countries, with an overall incidence of 47.6 cases per 100,000 population [1]; in Canada, with 36.1 cases every 100,000 person-years [2]; and the third most important bacterial foodborne diseases in the US [3]. *Campylobacter *spp. are found still at high prevalence in retail broiler carcasses in the US [4; 5], and the isolation of *Campylobacter *spp. from clinical and food samples has always been done using microaerobic conditions, generally 85% N_2_, 10% CO_2 _and 5% O_2_, during the enrichment of the samples and during the incubation of plate media. Different methods have been developed to generate microaerobic atmospheres and for a small number of samples, sachets that generate CO_2 _are commonly used [6]. If a larger number of samples are processed weekly, the evacuation-replacement is a more economical alternative. In this system, the air in the jar is partially removed by a vacuum pump and then replaced with a microaerobic gas mix. For a large number of samples, or to create unique microaerobic gas mixes with increased H_2 _content, more sophisticated microaerobic workstations have been developed [7].

Besides generating microaerobic conditions, several O_2_-quenching agents have been traditionally added to enrichment broths and agar plates for the isolation of *Campylobacter *spp. These agents neutralize the toxic effects of oxygen radicals and include blood or alkaline hematin [8; 9], charcoal [10], iron salts and norepinephrine [11], and ferrous sulfate, sodium metabisulfite and sodium pyruvate (known as FBP supplement) [12]. In general, if blood or charcoal is added to agar plates, no other O_2 _quenching compounds are added [9]. To ensure the microaerobic gas mix for the length of incubation (at least 48 h) sealed jars are commonly used, although plastic bags utilized to freeze food products with a "ziplock" type closing to prevent air leaks have been successfully used with gas-generating sachets and manual evacuation-replacement systems [13; 14].

Although a microaerobic mix is indispensable to grow *Campylobacter *spp. on agar plates, we have long suspected that no extra addition of any microaerobic gas mix is needed to keep *Campylobacter *spp. alive or even grow them in enrichment broths. In the present study we evaluated 108 retail broiler meat samples and compared the efficacy of Bolton broth incubated under microaerobic conditions using an evacuation-replacement system (subsamples M) versus incubation under aerobic conditions (subsamples A) for the isolation of naturally occurring *Campylobacter *spp. Presumptive *Campylobacter *spp. collected on agar plates were confirmed and identified with multiplex polymerase chain reaction (mPCR) assays and their DNA relatedness was analyzed using pulsed-field gel electrophoresis (PFGE). In addition, enriched broth cultures were analyzed with ribosomal intergenic spacer analysis (RISA) and denaturing gradient gel electrophoresis (DGGE) to determine the variability in the total bacterial population profiles of enrichment broths from subsamples M and A. Our results indicate that microaerobic conditions that allow *Campylobacter *spp. to grow are naturally created in enrichment broths without the addition of extra microaerobic gas mix, and therefore a simplified method has been developed to identify these bacteria in food samples.

## Results

### Similar number of *Campylobacter *positive subsamples

**Figure 1:**
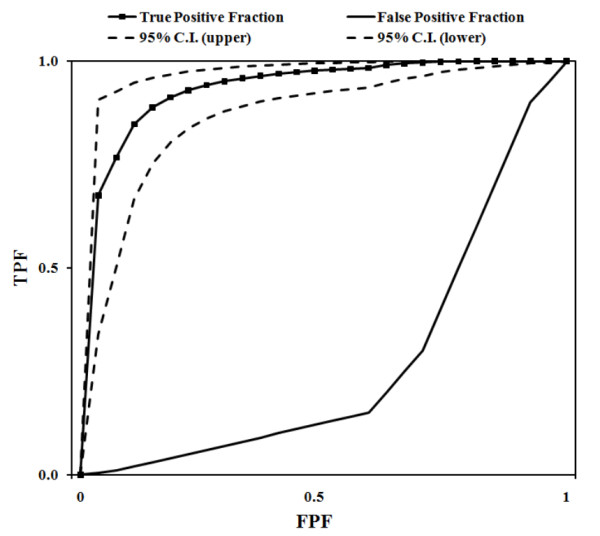
**ROC curves**. A high true positive fraction is shown with the upper and lower 95% confidence interval values. Consistent results were obtained from subsamples M (microaerobic conditions) and subsamples A (aerobic conditions) indicating that both methods were equivalent to isolate *Campylobacter *spp. from retail broiler meat.

From 108 retail broiler meat samples analyzed for the presence of *Campylobacter *spp., 48 (42%) were positive from the microaerobic subsamples (subsamples M), and 46 (44%) were positive from the aerobic subsamples (subsamples A). Combining the data from subsamples M and A resulted in a total of 56 (52%) positive samples for *Campylobacter *spp. Statistical comparison by chi-square showed that the number of *Campylobacter *positives from subsamples M and A were similar (*P *> 0.05), even when analyzing the subsamples by product (breasts or thighs) (Table 1). The sensitivity, specificity and accuracy were high (0.78 or above), and the Kappa values were above 0.50 for all comparisons, with the observed agreement in the Kappa value (considered the best agreement) always above 0.7 [15]. These high values reflected the large number of samples that were either positive (38 samples) or negative (52 samples) in both subsamples M and A, as calculated by 2-by-2 tables (data not shown). Receiver operating characteristic (ROC) curves also showed that the true positive fraction was high and within the 95% confidence interval calculated for this dataset (Figure 1).

**Table 1:** Number of subsamples M and A that were positive for *Campylobacter *spp.

	*Campylobacter *Positive (%)		
	
Enrichment Conditions	Breast	Thighs	Total
Microaerobic	20 (38)	28 (45)	48 (44)
Aerobic	18 (34)	28 (45)	46 (43)
Statistics			
χ^2 a^	0.10	0.00	0.50
P value	0.75	1.00	0.81
Sensitivity	0.81	0.88	0.79
Specificity	0.78	0.85	0.87
Accuracy	0.80	0.86	0.83
Kappa value	0.58	0.73	0.66

^a ^A chi-square values ≤ 3.84 assumes the null hypothesis that means from the reference method (microaerobic conditions) are equivalent to means from the test method (aerobic conditions) and cannot be rejected at the 5% level of confidence (*P *< 0.05).

### mPCR assays identified both *C. jejuni *and *C. coli *species

Table 2 shows the number of isolates collected and identified from subsamples M and A, and for each product type. A 100% agreement was found between the mPCR assay described in Materials and Methods and the mPCR extensively used in our laboratories [16; 17]. All *Campylobacter *isolates were confirmed as either *C. jejuni *or *C. coli*, with *C. jejuni *comprising 83% and 85% of the isolates for subsamples A and M, respectively. In 32 samples, subsamples M and A had *C. jejuni*, while six samples yielded *C. coli *in both subsamples. In 18 samples, only one of the subsamples (either M or A) was positive for *Campylobacter*.

**Table 2:** Speciation of *Campylobacter *isolates using the mPCR assay described in Material and Methods and a previously described mPCR assay [17].

		*C. jejuni*		*C. coli*	
		
Enrichment Conditions	Total (%)	Breast	Thighs	Breast	Thighs
Microaerobic (subsamples M)	48 (44)	19	22	1	6
Aerobic (subsamples A)	46 (43)	16	22	2	6

### PFGE similarity was high for most isolates collected from subsamples M and A

**Figure 2:**
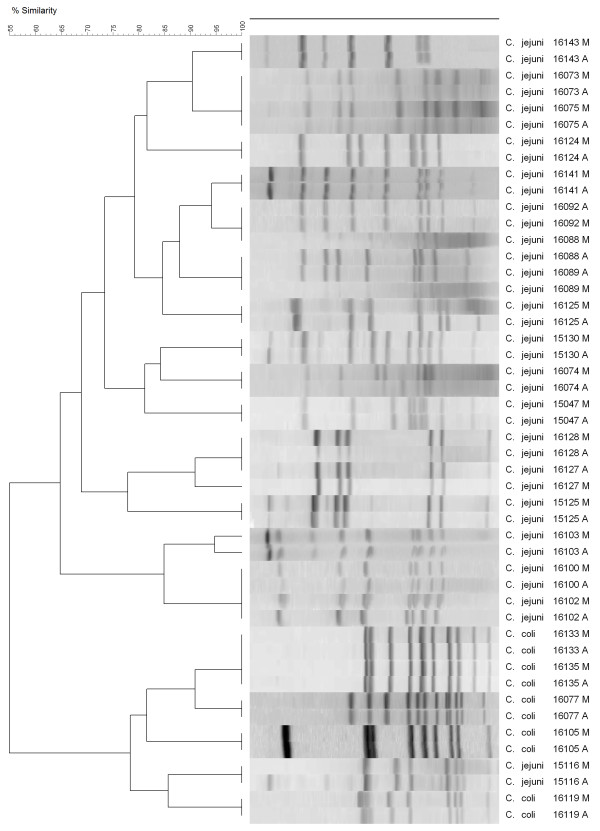
**PFGE results**. Isolates collected from subsamples M showing a high degree of similarity (> 90%) to isolates collected from subsample A. Pairwise comparisons were done using the Dice correlation and clustering analyses with the unweighted pair group mathematical average (UPGMA) clustering algorithm of BioNumerics ver. 5 (Applied Maths, Austin, TX, USA). The optimization tolerance was set at 2% and the position tolerance for band analysis was set at 4%.

**Figure 3:**
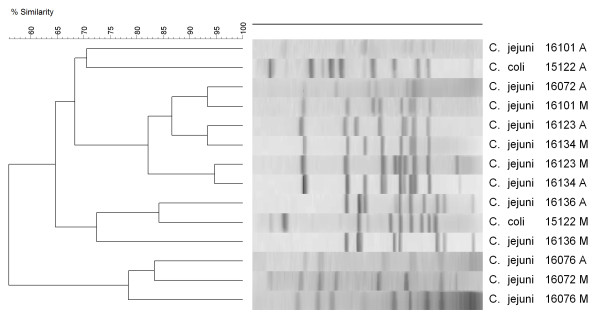
**PFGE results**. Isolates collected from subsamples M showing a low degree of similarity (< 90%) to isolates collected from subsample A. Pairwise comparisons and cluster analyses were done as described in Figure 2.

PFGE analysis of 48 isolates (24 samples) showed a high genomic DNA relatedness between strains from subsamples M and the corresponding isolates from subsamples A (Figure 2). For 14 isolates (7 samples), the similarity between isolates from subsamples M and A was lower than 90% (Figure 3).

### Bacterial diversity measured by RISA and DGGE studies vary considerably among samples and subsamples

**Figure 4:**
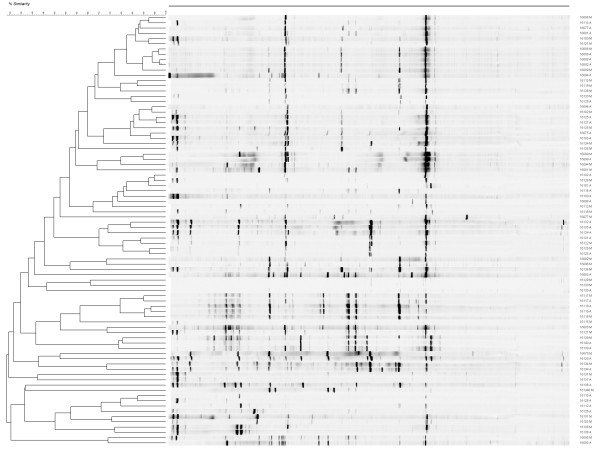
**Results from RISA analysis**. A low percentage of DNA similarity was found between the DNA profiles from subsamples M and the DNA profiles from subsamples A.

The results from the ARISA analysis of 41 subsamples M and 41 complimentary subsamples A, chosen at random, showed a large variation in the microbial community and a lack of similarity patters intra- or inter-sample (Figure 4). Similar results were found using BioNumerics and the Pearson correlation to compare the band patterns of subsamples M and A by DGGE. Even when analyzing the data using the Dice coefficient, which takes into account band migration, the results from subsamples M and A showed low DNA similarity at a cutoff point of 90% (data not shown). Table 3 shows the nearest neighbor identified from a BLASTn comparison of DGGE band sequences from subsamples M and A. Sequencing information suggested that the bacteria present in most subsamples were facultative anaerobes and microaerobic organisms. BLAST results indicated a high degree of similarity of some rDNA amplicons (> 90%) with *Acinetobacter *sp., *Campylobacter jejuni*, *Lactobacillus *sp. and *Pseudomonas *sp., and lower identity (80-90%) with *Lactobacillus *sp. and uncultured bacterial species. The sample that showed the largest bands separation had five well-distributed DNA bands in the gel (Table 3 A through E; DGGE bands K1 through K5). This sample was used consistently in DGGE gels as marker to normalize the gels and to allow for gel-to-gel comparisons using BioNumerics. A BLAST comparison showed that the sequences from these bands were similar to *Acinetobacter *sp. and *Lactobacillus *sp. (Table 3).

**Table 3:** Results from BLAST analysis of sequenced DGGE bands.

Marker	Band ID	BLAST nearest homology (GenBank accession number)	% Identity
A	K 1	*Acinetobacter *sp. (FN563421)	96
B	K 2	Uncultured Myxococcales bacterium (FJ435015)	93
C	K 3	*Lactobacillus *sp. L21 (AF159000)	87
D	K 4	*Lactobacillus *sp. (FJ971864)	95
E	K 5	*Lactobacillus *sp. JN4 (AF157041)	90
Microaerobic	subsample^a^	*Campylobacter jejuni *(GQ479820)	98
		*Lactobacillus *sp. 30A (FJ971864)	98
		*Pseudomonas *sp. CB10 (EU482914)	98
		*Pseudomonas *sp. R-35702 (AM886093)	97
Aerobic	subsample^a^	*Campylobacter jejuni *(GQ479820)	98
		*Lactobacillus *sp. JN4 (AF157041)	83
		*Pseudomonas *sp. CB11 (EU482915)	98
		Uncultured bacterium clone FF_e08 (EU469596)	

Marker bands were used in all the gels.

^a ^Unique DGGE bands from each subsample.

### O_2 _content decreased during the incubation of enrichment broths

**Figure 5:**
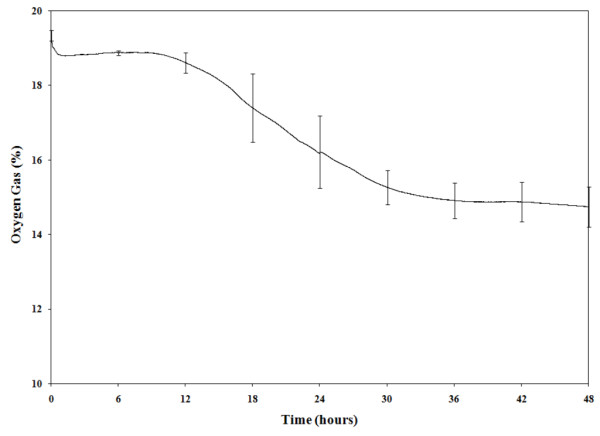
**Oxygen measurements**. Percentage of O_2 _in the head space of plastic bags throughout 48 h of incubation at 42°C. Average ± SEM of six measurements from subsamples positive for *Campylobacter *spp. after incubation under aerobic conditions. Measures were taken with an O_2 _sensor (Vernier, Beaverton, OR) as the percentage of O_2 _in the air in the head space.

**Figure 6:**
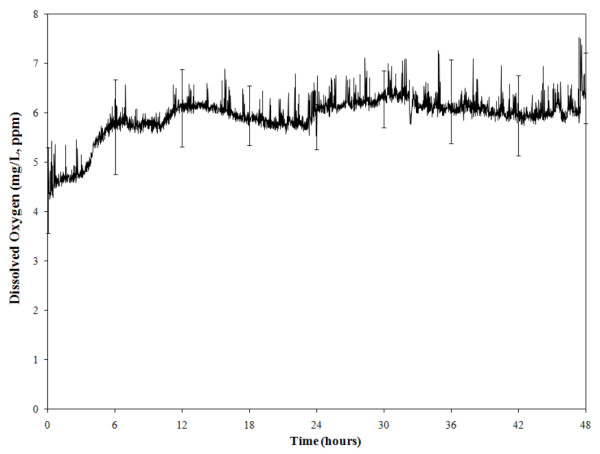
**Oxygen measurements**. Amount of dissolved oxygen (DO) in ppm in the enrichment broth. DO was measured at 1 inch from bottom of the bags, throughout 48 h of incubation at 42°C. Average ± SEM of six measurements from subsamples positive for *Campylobacter *spp. after incubation under aerobic conditions. Measurements were taken with a dissolved oxygen sensor (Vernier) and amount of oxygen in the liquid was recorded as mg/l or ppm.

In samples incubated in Bolton broth without the addition of any microaerobic gas mix, the amount of O_2 _in the head space of the bags decreased over time and was at or below 17% at 24 h of incubation. The amount of O_2 _in the atmosphere was stable between 14 and 16% by 30 h of incubation; however, the amount of O_2 _never reached less than 14% (Figure 5). The amount of dissolved O_2 _in the enrichment broth, measured one inch from the bottom of the enrichment bags, reached 6 ppm at around 6 h of incubation. This value was stable thereafter and never reached above 7.5 ppm (Figure 6). The presence of naturally occurring *Campylobacter *spp., either *C. jejuni *or *C. coli*, did not alter any of the values obtained with the sensors. In addition, incubation of 100 ml of Bolton broth without meat samples and without the addition of blood resulted in a similar pattern of DO values. In samples in which the O_2 _sensors were double bagged and gassed with a microaerobic gas mix, the DO decreased to around 5 ppm and remained stable for up to 72 h (data not shown). Identical patterns of dissolved O_2 _levels were found when using ziplock plastic bags commonly used to freeze food products (The Glad Products Company, Oakland, CA) (data not shown).

## Discussion

Several methods have been developed to generate microaerobic conditions for the growth and multiplication of *Campylobacter *spp. These methods are routine and are consistently used during the enrichment of food samples or during the incubation of inoculated plate media. However, little is known about the actual changes in O_2 _content in enrichment broth media during incubation (37°C or 42°C). Our experiments were aimed at determining the changes of O_2 _content in the broth and in the air of the head space of the bags used to enrich the samples for the isolation of *Campylobacter *from retail broiler meat. The premises of this work was that the incubation of enrichment broth may naturally create microaerobiosis conducive to the grow of *Campylobacter *spp. Samples were therefore divided in two subsamples which were in turn incubated under microaerobic conditions (M) or aerobic conditions (A).

We used an unpaired sample design, where the enrichment conditions differ between the reference (subsamples M) and the alternative method (subsamples A), and confirmed all presumptive positives using the same molecular protocols. Because the comparison of two qualitative methods is best accomplished near the limit of detection of these methods, we used naturally contaminated broiler meat samples, which have the lowest contamination that can be naturally found [4; 17]. The statistical analyses of data from unpaired samples are performed in the same way as for paired samples, mainly using McNemar's chi square test [18]. The number of *Campylobacter *positive subsamples was statistically similar between subsamples M and A, and all isolates were clearly identified as *C. jejuni *or *C. coli*. These results demonstrate that enrichment broths incubated under normal, aerobic conditions are sufficient to detect *Campylobacter *spp. in retail broiler meat. There was an increase in number of total positive samples by 10% when combining the result of the two subsamples. These findings have been already reported several times for commercial broiler meat naturally contaminated with *Campylobacter *spp. [4; 17]. In addition, a ROC curve of the data showed a high true positive fraction, or rate, and a very low false positive fraction, which indicated a very strong correspondence in the results between the reference (subsamples M) and the alternative methods (subsamples A).

The traditional methodology of enriching 25 g of meat is the one suggested by the Bacteriological Analytical Manual of the Food and Drug Administration (FDA) [19], the Microbiology Laboratory Guidebook of the Food Safety and Inspection Services of the U. S. Department of Agriculture (FSIS UDSA) [20], the International Organization for Standardization [21], the Health Protection Agency of the UK [22], and several other countries' regulatory agencies. However, this methodology does not appear to be optimized to detect the true prevalence of *Campylobacter *spp. in retail broiler meat. PCR analysis of the isolates showed that *C. jejuni *or *C. coli *species are the only *Campylobacter *spp. found in retail broiler meat. Some samples can be contaminated with both species [17] but again the current methodology used in food samples is not accurate enough to reveal the extent of contamination of the same product with different *Campylobacter *strains. PFGE analysis further demonstrated that a single meat sample could be contaminated with two, or maybe more, isolates from the same species. For all practical purposes, *C. jejuni *and *C. coli *are the only two *Campylobacter *spp. found in retail poultry meat because no *C. lari *has been identified since the introduction of molecular techniques for routine identification of *Campylobacter *isolates, approximately 15 years ago [23]. The data collected with the O_2 _sensors showed that the amount of O_2 _in the enrichment broth was stable around 5-7 ppm after 6 h of enrichment. These O_2 _levels can be obtained by pressing out the air before closing the sample bags, and without the need of any vacuum, as is required when removing the air from a hard container. Whirl-Pak or ziplock bags performed similarly, showing that they are impervious to changes in the air trapped inside [13]. The fact that bags with only the enrichment broth (without meat or blood) created microaerobic conditions has encouraged us to continue this line of research, and we are currently testing other broths without blood to isolate *Campylobacter *spp. from retail broiler meat. Therefore, an inexpensive, simplified method can be developed for routinely use in the isolation and detection of *Campylobacter *spp. from food products.

Incubation of broth under normal aerobic conditions, with or without airspace, was done in the early 1980s to isolate *Campylobacter *spp. from fecal samples [24], and the use of 10% O_2_, 10% CO_2 _and 80% of N_2 _facilitated and sustained the growth of *Campylobacter *spp. [25]. The ISO normative 10272-1:2006 requires a microaerobic environment but provides for an alternative incubation in a microaerobic atmosphere created by "screw-capped bottles or flasks filled with enrichment broth, leaving a headspace of less than 2 cm, and tightly closing the caps" [21]. But much has been speculated about the need to have a higher surface area of the meat samples during enrichment to yield a higher number of positive samples under microaerobic conditions [26], or the exact depth of the airspace, the appropriate ratio of air to broth [27], and the correct type of incubation container to promote the growth of *Campylobacter jejuni *[28] to avoid significant difference in the results if a microaerobic atmosphere is not used [27]. Therefore, the microaerobic conditions are routinely used to isolate *Campylobacter *spp. However, our results do not suggest any correlation between surface and microaerobic conditions and do not support the notion that air to broth ratio and the type of container are indispensable to isolate *Campylobacter *spp. Our results point to the simple fact that any closed plastic bag naturally produces microaerobic environments conducive to the growth of *Campylobacter *spp. without the need to add any microaerobic gas mix. In our experiments, bags were closed to leave a minimum airspace and the samples were mixed, without stomaching, for few seconds. Thus, bags with subsamples M had the same contact surface as bags with subsamples A.

The microbial population of the enriched samples in Bolton broth, as assessed by RISA and DGGE, was diverse. There are no current data on the microbial assemblage of retail broiler meat as a predictor to the presence of a bacterial pathogen, such as *Campylobacter*. Most of the work on the bacterial community of broiler meat was done more than 20 years ago using direct bacterial counts, and very few research studies have used culture-independent methods to study the microbial profile of these foods [29]. It is known, however, that some cold-tolerant bacteria, such as *Enterobacteriaceae*, *Acinetobacter *and *Pseudomonas*, are commonly present on broiler meat [30]. These bacteria are primarily facultative anaerobes or microaerobic organisms, and the ribosomal RNA gene sequences recovered in our samples, especially form the most prominent bands from DGGE gels, had a high similarity to these bacterial groups.

RISA and DGGE can be used to broadly characterize the total microbial population in complex samples. The results from these techniques were analyzed using the Pearson correlation, which is the standard procedure for comparison of densitometric curves [31; 32]. We analyzed the results with the Pearson correlation and also the Dice coefficient, which takes into account only the band position and not the band thickness, as it is the case in densitometric curves. Although the Dice correlation showed a higher DNA relatedness among corresponding M and A subsamples, the variability in the bacterial populations in each set of subsamples was still large and appeared to be more attributable to the original bacterial composition of the sampled meat itself than to the enrichment conditions (aerobic vs. microaerobic). A significant limitation of DGGE-derived phylogenetic data with the primers used in this study is the relatively short rDNA sequence obtained from each amplicon, thereby reducing the degree of phylogenetic inference that may be assigned to each band. Yet, both RISA and DGGE produced consistent results regarding the variability in the bacterial assemblages associated with retail broiler meat samples.

## Conclusions

In summary, our results indicated that the enrichment of retail broiler meat at 42°C in closed plastic bags and without the addition of a microaerobic mix is adequate for the isolation of *Campylobacter *spp. With the advancement of DNA-based biosensors and automation for bacterial detection, enrichment broths could be screened for the presence of *Campylobacter *spp. in a shorter time, with greater sensitivity and without the generation of any microaerobic condition. In addition, food microbiology laboratories interested in establishing techniques for the isolation of *Campylobacter *from retail meat will have access to a cost-effective enrichment procedure without the need to invest in systems to generate microaerobiosis. Reference documents from the FDA and FSIS USDA should eventually be updated to provide for an alternative, simplified protocol that yields similar number of *Campylobacter *positive samples as the current reference protocols.

## Methods

### Sample preparation, incubation and *Campylobacter *isolation

Retail broiler meat samples (total = 108 samples; 49 breasts and 59 thighs) were purchased from local stores (Auburn, AL) from April 2009 to October 2010. Samples were tested in batches of three to five samples per week. Each meat package was considered one sample, and from each package ~1-inch pieces were cut aseptically and mixed thoroughly. For all samples, 25 g of meat was weighed two times (two subsamples) in individual, sterile Whirl-Pak^® ^(Nasco, Fort Atkinson, WI). Each subsample was enriched in 100 ml of Bolton's broth (with antimicrobial supplements) and 5% (v/v) of lysed horse blood [17]. The control subsamples (microaerobic subsamples) were incubated in anaerobic jars gassed with a microaerobic gas mix (85% N_2_, 10% CO_2_, 5% O_2_; Airgas, Radnor, PA) using the evacuation-replacement system MACSmics Jar Gassing System (Microbiology International, Frederick, MD). The other subsamples (aerobic subsamples) were incubated without the addition of microaerobic gas mix, by closing the bags after removing the remaining air manually. All subsamples were incubated at 42°C for 48 h.

After incubation and for all subsamples, 0.1 ml of the enriched broth was transferred to modified charcoal cefoperazone deoxycholate agar [10] through a 0.65 μm membrane filter as described elsewhere [33]. All agar plates were incubated under microaerobic conditions at 42°C for 48 h. Presumptive *Campylobacter *colonies were observed under phase contrast microscopy (Olympus BX51, Olympus America Inc., Center Valley, P) for spiral morphology and darting motility. Presumptive isolates were stored at -80°C in tryptic soy broth (Difco, Detroit, MI) supplemented with 20% glycerol (v/v) and 5% (v/v) lysed horse blood for further analysis.

### Identification of presumptive *Campylobacter *isolates by mPCR assays

*Campylobacter *isolates were recovered from frozen stocks by transferring to Brucella agar plates supplemented with 5% horse blood and through 0.6 μm membrane filters as described above. Plates were incubated at 42°C under microaerobic conditions for 24 h. Bacterial DNA was extracted using the Wizard^® ^Genomic DNA Purification Kit as described by the manufacturer (Promega, Madison, WI), but bypassing the RNA digestion step. Isolates were identified with a previously described mPCR assay [17; 34; 35], and a newly developed mPCR comprised of two sets of primers, one targeting the *glyA *gene of *C. jejuni *and the other targeting the *ask *gene of *C. coli*. Gene sequences downloaded from NCBI GenBank were aligned and analyzed using Molecular Evolutionary Genetics Analysis (MEGA) software [36] and primers were designed with the Integrated DNA Technologies PrimerQuest software. (Integrated DNA Technologies http://www.idtdna.com) The sequences of the primers are shown in Table 4. *C. jejuni *ATCC (American Type Culture Collection) 700819 and *C. coli *ATCC 43473 were used as control strains to set up the PCR conditions. The annealing temperatures of these primers were optimized with a gradient PCR program of a DNA ENgine^® ^Thermal Cycler (Bio Rad laboratories, Hercules, CA), and the final conditions for this mPCR assay were 20 cycles of 94°C for 30 seconds; 63°C for 1 minute and 72°C for 1 minute. Amplified products were detected by standard gel electrophoresis in 1.5% agarose (Ultra Pure DNA Grade Agarose, Bio-Rad Laboratories) in tris-borate-EDTA buffer at 100 V for 40 minutes. DNA bands in the gels were stained with ethidium bromide and visualized using a VersaDoc™ Imaging System (Bio-Rad Laboratories).

**Table 4:** Primers developed in this study for the specific identification of *C.jejuni *and *C. coli*.

Target Gene	Primer Name	Sequence (5'-3')	Tm (°C)	G+C Content (%)	Product Size (bp)
*glyA *	F-JK	TGGCGGACATTTAACTCATGGTGC	59.6	50	264
	R-JK	CCTGCCACAACAAGACCTGCAATA	59.5	50	
*ask *	F-JK	GGCTCCTTTAATGGCCGCAAGATT	59.8	50	306
	R-JK	AGACTATCGTCGCGTGATTTAGCG	58.5	50	

### Typing of *Campylobacter *isolates with PFGE

Isolates from 31 samples for which both subsamples were positive were randomly selected for PFGE analysis. *Campylobacter *isolates were typed using pulsed-filed gel electrophoresis (PFGE) following previously described protocols [16; 23]. Briefly, DNA was digested with *Sma*I and separated using a CHEF DR II system (Bio-Rad Laboratories, Hercules, CA) on 1% agarose gels (SeaKem Gold agarose; Lonza). The DNA size marker used in the gels was *Salmonella enterica *subsp. *enterica *serovar Braenderup strain H9812 (ATCC BAA-664) restricted with *Xba*I. Restriction enzymes were purchased from New England BioLabs (Ipswich, MA). Gels were stained and visualized as described above (mPCR assays) and TIFF images were loaded into BioNumerics version 6 (Applied Maths, Austin, TX) for analysis. Pairwise-comparisons were done with the Dice correlation coefficient, and cluster analyses were performed with the unweighted pair group mathematical average (UPGMA) clustering algorithm. The optimization and position tolerance for band analysis were set at 2 and 4%, respectively, and similarity among PFGE restriction patters was set at 90%.

### DNA extraction from enrichment broths for bacterial population analysis

DNA from enrichment broths after 48 h of incubation (subsamples M and A) was extracted using the Wizard^® ^Genomic DNA Purification Kit (Promega). To determine the microbial community profile of these subsamples, ribosomal intergenic spacer analysis (RISA) and denaturing gradient gel electrophoresis were performed (DGGE).

Forty-one sample sets chosen at random (22 negative for *Campylobacter *spp. in both subsamples and 19 positive for *Campylobacter *spp. in both samples [16 *C. jejuni*/*C. jejuni *and 3 *C. coli*/*C. coli*]) were analyzed by ARISA. RISA was generated by amplification of the internal spacer region (ISR) using the universal primers according to Cardinale et al. [37]. Amplified products were separated by electrophoresis on the NEN Global Edition IR2 DNA Analyzer (LI-COR, Lincoln, NE) following manufacturer's instructions. RISA images were processed with BioNumerics (Applied Maths). Following conversion, normalization, and background subtraction with mathematical algorithms, levels of similarity between fingerprints were calculated with the Pearson product-moment correlation coefficient (*r*). Cluster analysis was performed using the UPGMA algorithm.

DGGE was performed using universal primers 338F (containing a 5' G+C clamp) and 518R, which amplify a segment of the 16S rDNA gene [38; 39]. PCR amplification consisted of 30 cycles of 5 min of denaturation at 94°C, 1 min of annealing at 55°C, and 1 min of extension at 72°C. The DGGE system (Ingeny phorU, Netherlands) had a denaturing gradient comprised of urea and formamide ranging from 45% to 65% in vertical polyacrylamide gels. Gels were stained with ethidium bromide and visualized under a UV gel imager. As a standard marker for gel comparison, every DGGE gel had one lane containing a DNA marker that had five specific bands. DGGE banding patterns were analyzed using BioNumerics (Applied Maths). Pairwise comparisons and cluster analysis were performed with the Pearson correlation coefficient and the Dice coefficient, and the UPGMA algorithm, respectively. The band position tolerance was set at 3% and a cut off value of 90% was used to determine similarity between subsamples. Selected bands from DGGE gels were excised and amplified using primers 338F (without the G+C clamp) and 518R. Amplicons were purified using the Wizard^® ^SV Gel and PCR Clean-up System (Promega), and PCR products were sequenced with an ABI 3730 sequencer (Applied Biosystems, Foster City, CA) at Lucigen Corporation (Middleton, WI). Sequences were aligned with MultAlin [40] and the consensus sequences were compared to the GenBank database using BLAST http://blast.ncbi.nlm.nih.gov/Blast.cgi. The accession numbers of the sequences deposited in GenBank are GU250527 through GU250536.

### Detection of O_2 _changes during the incubation of enrichment broth

The changes in the amount of O_2 _in the enrichment broth and the head space in the enrichment bags was measured in eight aerobic subsamples using a dissolved oxygen (DO) sensor (amount of oxygen in liquid measured as mg/l or ppm), and an oxygen (O_2_) sensor (percentage of oxygen in the air). These sensors were purchased from Vernier (Beaverton, OR). A double bagging system was used to avoid air leaks during the measurements taken with the O_2 _sensors during incubation. Changes in O_2 _concentration were measured in all subsamples. The O_2 _Gas Sensor was calibrated to the environment within the plastic bag which produces condensation (100% humidity), and therefore was started at 20.1 O_2 _in percentage by volume. The DO sensor was positioned in the enrichment bag with the collection tip of the sensor placed at the bottom of the enrichment broth with the subsample. The O_2 _sensor was placed in the head space of the bag above the liquid. The excess air was expelled from the bag before sealing and incubation for 48 h. The DO sensor was calibrated by pre-warming the probe for 10 min in the broth before starting the readings. Throughout incubation, the sensors were connected to a laptop computer with the Logger Lite™ data collection program (version 1.4) that recorded readings every 1 min. The data were analyzed using Microsoft Excel (Microsoft Corporation, Redmond, WA).

### Statistical analyses

An unpaired sample design was used where the number of *Campylobacter *positive subsamples enriched under microaerobic conditions (reference method) was compared to the number of *Campylobacter *positive subsamples enriched under aerobic conditions (alternative method). Statistical comparisons were made using the formula mcnemar. test (x, y, correct = TRUE) of R [41], which is the McNemar's chi-squared (γ^2^) test for count data, and it is based on McNemar's Test for correlated proportions [42]. The accuracy, sensitivity, specificity, and Kappa values for the test were calculated using 2-by-2 tables according to Hanrahan and Madupu [43]. A receiver operating characteristic (ROC) curve was determined with a web-based calculator with an ordinal rating scale of 1 through 4, where 1 represents samples that were negative for *Campylobacter *spp. in both subsamples, and 4 represents samples that were positive for both subsamples [44].

## Authors' contributions

PZ carried out the sample collection, the DNA preparation, PFGE and PCR-DGGE assays, and image statistical analysis. SKH helped with sample collection and DGGE analysis. ML helped optimize the DGGE analysis. CRA carried out RISA assays. SB helped analyze data and wrote part of the manuscript. JRK carried out the primer design to differentiate *C. jejuni *from *C. coli*. OAO conceived and coordinated the study, designed and revised the manuscript. All authors read and accepted the final version of the manuscript.
